# Validation of a Pharmacist-Led Semaglutide Prescribing Algorithm for Type 2 Diabetes and Chronic Kidney Disease: Research Letter

**DOI:** 10.1177/20543581261476643

**Published:** 2026-08-13

**Authors:** Maneka Sheffield, Emma Phelan, Natalie Ratajczak, Marisa Battistella, Karthik Tennankore, Ayodele Odutayo, Steven Soroka, Penelope Poyah, Keigan More, Heather Naylor, Natalie Kennie-Kaulbach, Daniel Rainkie, Jo-Anne Wilson

**Affiliations:** 1 Faculty of Health, College of Pharmacy, 3688 Dalhousie University, Halifax, NS, Canada; 2 Kidney Research Institute of Nova Scotia, Nephrology, Nova Scotia Health, Halifax, NS, Canada; 3 Leslie Dan Faculty of Pharmacy, 7938 University of Toronto, Toronto, ON, Canada; 4 Department of Nephrology, Toronto General Hospital, Toronto, ON, Canada; 5 Department of Pharmacy, 7989 University Health Network, Toronto, ON, Canada; 6 Faculty of Medicine, 3688 Dalhousie University, Halifax, NS, Canada; 7 Division of Nephrology, NSH, Halifax, NS, Canada; 8 Faculty of Medicine, University of Toronto, Toronto, ON, Canada; 9 Sunnybrook Health Sciences Center, Toronto, ON, Canada; 10 Pharmacy Department, 10068 Horizon Health Network, St. John, NB, Canada; 11 Nova Scotia Health Research and Innovation, 432234 Nova Scotia Health, Halifax, NS, Canada

**Keywords:** algorithm validation, semaglutide, pharmacist, guideline-directed prescribing, diabetic kidney disease, primary care

## Abstract

Diabetes is a leading cause of chronic kidney disease, yet gaps in the use of guideline-directed therapies persist in primary care. In 2025, our group developed and validated community pharmacist-led prescribing algorithms for individuals with an estimated glomerular filtration rate ≥30 mL/min/1.73 m^2^, targeting angiotensin-converting enzyme inhibitors or angiotensin receptor blockers, sodium-glucose cotransporter-2 inhibitors, and nonsteroidal mineralocorticoid receptor antagonists. To address the evolving therapeutic landscape, we developed and validated a semaglutide-specific algorithm to complement this approach. Algorithm development and validation followed Lynn’s method. A two-part questionnaire per algorithm item assessed content and face validity, with nephrology clinicians (nephrologists and kidney pharmacists) and community pharmacists rating items using Likert scales. Content validity was measured using item-level (I-CVI) and scale-level (S-CVI/Ave) indices, while face validity was assessed by level of agreement to five statements per round. The algorithm was iteratively revised between rounds. Ten nephrology clinicians (five per round for three rounds) and 12 community pharmacists (six per round for two rounds) participated. For clinicians, the I-CVI ranged from 0.6 to 1.0, with an overall S-CVI/Ave of 0.89 across three rounds. Among pharmacists, all items met the prespecified content validity threshold for both rounds (I-CVI ≥0.83; P < 0.05). Face validity exceeded the 70% consensus threshold for both validator groups. This study adds a validated semaglutide-specific algorithm to our existing pharmacist-led care model, expanding a suite of algorithms to support uptake of disease-modifying therapies for individuals with type 2 diabetes and chronic kidney disease in primary care. Implementation and evaluation in Nova Scotia community pharmacy clinics are underway.

## Introduction

Type 2 diabetes (T2D) is a major contributor to chronic kidney disease (CKD) and kidney failure in Canada.^1^ As kidney function declines in individuals with diabetic kidney disease (DKD), the risk of adverse cardiovascular outcomes, including heart failure, hospitalization, and premature mortality increases, adding to healthcare burden.^2^

Advances in pharmacotherapy have introduced several guideline-directed medical therapies (GDMT) with proven cardiorenal benefits for individuals with T2D and CKD. These include sodium-glucose cotransporter-2 inhibitors (SGLT2i), nonsteroidal mineralocorticoid receptor antagonist (nsMRA), and glucagon-like peptide-1 receptor agonist (GLP-1RA). Guidelines recommend the use of these agents alongside lifestyle modification, glycemic and blood pressure optimization, and use of angiotensin-converting enzyme inhibitors (ACEi) or angiotensin receptor blockers (ARB).^3,4^ Despite strong evidence supporting the benefits of GDMT, uptake remains low in primary care, where many individuals with early-categories of CKD are managed.^5,6^

Community pharmacists are increasingly recognized for their role in chronic disease management in primary care. In many Canadian provinces, expanded scope of practice allows pharmacists to assess patients, order laboratory tests, and prescribe medications. In Nova Scotia, community pharmacy primary care clinics provide pharmacist-led services, particularly for individuals without access to a primary care provider. In 2025, our group published the development and validation of medication algorithms for DKD, covering ACEi or ARB, SGLT2i, nsMRA, designed to support community pharmacists in optimizing evidence-based prescribing.^7^ Since then, emerging evidence has established the GLP-1RA, semaglutide, as another key therapy for individuals with T2D and CKD. Building on our prior work, this study develops and validates a semaglutide-specific algorithm to support safe, consistent therapy initiation and management in individuals with T2D and CKD with an estimated glomerular filtration rate (eGFR) 30–60 mL/min/1.73 m^2^.

## Methods

The development and validation of the semaglutide algorithm were conducted in two phases. Phase 1 involved the development of the algorithm followed by iterative revisions based on feedback from nephrology clinicians. Phase 2 consisted of formal validation of the algorithm by both nephrology clinicians and community pharmacists. This study received approval from the Nova Scotia Health Research Ethics Board (File #1030369).

### Phase 1: Development of Semaglutide Algorithm

Comprehensive searches of Ovid MEDLINE, Embase, CINAHL, and the Cochrane Library (2016–2026) were conducted to inform semaglutide algorithm development, with findings summarized in Supplemental File 1.

An evidence- and expert-informed semaglutide algorithm was developed using Lynn’s three-step method: domain identification, item generation, and instrument formation.^8^ Domains mirrored our previous study (screening and assessment, prescribing, laboratory monitoring), and items were drawn from literature, guidelines, and product monographs. A sequential algorithm was constructed and refined in a development meeting with five nephrology clinicians.

### Phase 2: Nephrology Clinician and Community Pharmacist Validation of Semaglutide Algorithms

Nephrology clinicians (nephrologists and pharmacists) and community pharmacists working in a community pharmacy primary care clinic who largely participated in the previous validation study were invited to participate.

Validation of the algorithm was conducted using an approach outlined by Lynn’s method.^8^A two-part questionnaire was developed to validate the algorithm’s content (e.g. appropriateness, accuracy, completeness of content) and face validity (e.g. clarity, comprehensibility, and appropriateness to the user) (Supplementary File 2). The algorithm was divided into three items (A-C) to align with the questionnaire (see Supplementary File 3). Nephrology clinicians and community pharmacist participants were emailed the questionnaire and algorithm to complete.

For *content validation,* participants rated the algorithm using a four-point Likert scale (1 = strongly disagree/unacceptable – Remove; 2 = disagree/unacceptable – major revision; 3 = agree/acceptable – minor revision; 4 = strongly agree – acceptable as is). Written comments were requested for suggested revisions. Any item receiving a rating of 1 or 2 from at least one participant was revised prior to the subsequent round.

The content validity index (CVI) was calculated using an item-level CVI (I-CVI) and scale-level CVI (S-CVI/Ave) based on the average method for each round.^8^ The I-CVI is the proportion of participants giving the item (A to C) a rating of 3 or 4. According to Lynn and others, for 6 participants in a round, at least 5 must agree (rating of 3 or 4) to establish content validity at p< 0.05 (I-CIV of ≥0.83).^8^ An item is considered invalid if 2 out of the 6 participants per round provide a rating of 1 or 2 (I-CVI ≤0.67). For a panel of 5 participants in a round, all must rate the item 3 or 4 to establish content validity at P<0.05 (I-CVI of 1)^8^. The S-CVI/Ave is the average of all I-CVI scores for all items (A to C) per round. Rounds continued until predefined validity consensus thresholds were met. We also calculated the overall I-CVI for each item and S-CVI/Ave across validation rounds for nephrology clinician and community pharmacist groups. An S-CVI/Ave ≥ 0.9 indicates that the algorithm has excellent content validity.

For *face validation*, participants rated statements about the algorithm on a five-point Likert scale assessing clarity, language, flow, usability, and recommendation for practice. Written feedback was permitted, and items rated 1–3 were revised. Percentages were calculated per statement, with ratings of 4 or 5 deemed valid. Consensus was defined as ≥70% agreement (ratings of 4 or 5)^7^. Components not meeting criteria were revised for subsequent rounds.

## Results

### Study Participants

The semaglutide algorithm was validated by ten nephrology clinicians across three rounds (five per round) from December 1, 2025, to January 10, 2026; half of the clinicians participated in two rounds. Clinicians were primarily from Nova Scotia, with three from New Brunswick and Ontario. Among nephrology clinicians, 50% were female with a mean±SD age of 47±10 and 18±9 years of experience. Twelve community pharmacists validated the algorithm in two rounds (six per round) between January 12 and February 12, 2026. The community pharmacists (all female) were drawn from urban (58%) and rural (42%) primary care pharmacy settings throughout Nova Scotia with a a mean ± standard deviation (SD) age of 46±9 years and 21±9 years of experience.

### Content and Face Validation

Over three rounds of nephrology clinicians, the I-CVI of the algorithm item (A-C) ranged from 0.6-1 (Table 1, Supplementary File 4). The overall S-CVI/Ave across three rounds was 0.89. The overall percentage of participants (averaged across three rounds) who agreed or strongly agreed to five face validity statements ranged between 87–93%. After nephrology clinician validation, the algorithm was validated by community pharmacists.

**Table 1.** Nephrology Clinician and Community Pharmacist Content and Face Validity Indices Scores: Algorithm to Assist Prescribing of Semaglutide for T2D in CKD

Content validation		
**Nephrology Clinician**		
**Round 1: Algorithm Item**	**I-CVI**	**S-CVI/Ave**
A	0.80	0.93
B	1	​
C	1	​
**Round 2: Algorithm Item**	**I-CVI**	**S-CVI/Ave**
A	0.60	0.73
B	0.60	​
C	1	​
**Round 3: Algorithm Item**	**I-CVI**	**S-CVI/Ave**
A	1	1
B	1	​
C	1	​
**Overall average (rounds 1-3)**	**I-CVI**	**S-CVI/Ave**
A	0.87	0.89
B	0.87	​
C	1	​
**Community Pharmacist**		
**Round 1: Algorithm Item**	**I-CVI**	**S-CVI/Ave**
A	1	1
B	1	​
C	1	​
**Round 2: Algorithm Item**	**I-CVI**	**S-CVI/Ave**
A	1	1
B	1	​
C	1	​
**Overall average (rounds 1-2)**	**I-CVI**	**S-CVI/Ave**
A	1	1
B	1	​
C	1	​

Face validation	
**Nephrology Clinician**	
**Statement**	**N (%) participants who agree or strongly agree**
The algorithm is clear and understandable	10 (87%)
The algorithm uses appropriate language and wording	10 (87%)
The algorithm flows in a logical manner	10 (93%)
I would use this algorithm in my own practice	10 (87%)
I would be confident recommending the use of this algorithm	10 (87%)
**Community Pharmacists**	
**Statement**	**N (%) participants who agree or strongly agree**
The algorithm is clear and understandable	12 (100%)
The algorithm uses appropriate language and wording	12 (100%)
The algorithm flows in a logical manner	12 (100%)
I would use this algorithm in my own practice	12 (100%)
I would be confident recommending the use of this algorithm	12 (100%)

I-CVI = item-level content validity index; S-CVI/Ave = scale-level content validity index based on average method; N= number.

Content validation was achieved after two rounds with pharmacist participants. The I-CVI of each item of the algorithm per round of validation met the threshold of 0.83 (p<0.05) for at least 6 participants (Table 1, Supplementary File 4). The calculated S-CVI/Ave over both rounds was 1, exceeding the threshold for content validity. The overall percentage of participants (averaged across 2 rounds) who agreed or strongly agreed to 5 face validity statements for the algorithm was 100%. Nephrology clinician and community pharmacist feedback resulted in revisions to the algorithm flow, enhanced patient education, and inclusion of precautions and monitoring for hypoglycemia, retinopathy, and frailty (Supplementary File 5). Supplemental File 6 presents the final validated semaglutide algorithm and its integration within all previously published algorithms.

## Discussion

In response to the evolving therapeutic landscape, we developed and validated a semaglutide-specific algorithm to complement our previously published medication algorithms for DKD.

Content validity was achieved across nephrology clinician and community pharmacist rounds (S-CVI/Ave 0.89 and 1), indicating the validators found the algorithm appropriate, accurate and complete. Face validity also exceeded the prespecified consensus threshold of 70%, showing that both clinicians and pharmacists considered the algorithm clear, comprehensive, and appropriate for use in practice.

Similar to our previously validated algorithms, the semaglutide algorithm provides a systematic, evidence- and nephrology-informed framework to support community pharmacists in prescribing and monitoring guideline-directed therapies for patients with an eGFR of 30–60 mL/min/1.73 m^2^, who are generally managed in primary care. Although many of these medications can be used at eGFR <30 mL/min/1.73 m^2^, this threshold was excluded because such patients are typically referred to and managed by a nephrologist. The algorithm includes checkpoints for monitoring response, providing education, and prompting referral to nephrology or collaboration with another prescriber.

Prior studies demonstrate that team-based, protocol-driven strategies involving pharmacists can improve access to and uptake of guideline-directed therapies in individuals with DKD.^9,10^ The DRIVE trial (Diabetes Remote Intervention to ImproVe Use of Evidence-Based Medications), a remote pharmacist-inclusive, team-based intervention, showed that protocol-driven care improves initiation of guideline-directed therapies in patients with T2D and kidney risk, supporting the role of pharmacists in addressing CKD care gaps.^9^ The CHAMP trial (Kidney Coordinated HeAlth Management Partnership), a cluster-randomized study, demonstrated that a population health management intervention, including a nephrology e-consultation, pharmacist-led medication management and patient education compared to usual care led to improved uptake of SGLT2 inhibitors and GLP-1RA.^10^ These findings highlight the potential of pharmacist-led approaches to optimize guideline-directed therapy and improve DKD care, particularly for those with limited access to primary care.

This study has similar strengths and limitations as our prior work,^7^ with the inclusion of previous community pharmacist participants, including some certified diabetes educators, enhancing reliability of algorithm validation. However, use of many of the same community pharmacists and clinicians as in the prior study may introduce bias. No endocrinologist was involved in validation, and all community pharmacists were female which may limit generalizability.

## Conclusion

We developed and validated a semaglutide algorithm with nephrology clinicians and community pharmacists involved in prior validation study of ACEi or ARB, SGLT2i and nsMRA. Engaging pharmacists in community pharmacy primary care clinics represents an innovative, population-level approach to address gaps in CKD prescribing and support guideline-directed care. Evaluation of these algorithms in Nova Scotia pharmacy clinics is currently underway.

## Supplemental Material

Supplemental Material - Validation of a Pharmacist-Led Semaglutide Prescribing Algorithm for Type 2 Diabetes and Chronic Kidney Disease: Research Letter Supplemental Material for Validation of a Pharmacist-Led Semaglutide Prescribing Algorithm for Type 2 Diabetes and Chronic Kidney Disease: Research Letter by Maneka Sheffield, Emma Phelan, Natalie Ratajczak, Marisa Battistella, Karthik Tennankore, Ayodele Odutayo, Steven Soroka, Penelope Poyah, Keigan More, Heather Naylor, Natalie Kennie-Kaulbach, Daniel Rainkie, Jo-Anne Wilson in Canadian Journal of Kidney Health and Disease.

Supplemental Material - Validation of a Pharmacist-Led Semaglutide Prescribing Algorithm for Type 2 Diabetes and Chronic Kidney Disease: Research Letter Supplemental Material for Validation of a Pharmacist-Led Semaglutide Prescribing Algorithm for Type 2 Diabetes and Chronic Kidney Disease: Research Letter by Maneka Sheffield, Emma Phelan, Natalie Ratajczak, Marisa Battistella, Karthik Tennankore, Ayodele Odutayo, Steven Soroka, Penelope Poyah, Keigan More, Heather Naylor, Natalie Kennie-Kaulbach, Daniel Rainkie, Jo-Anne Wilson in Canadian Journal of Kidney Health and Disease.

Supplemental Material - Validation of a Pharmacist-Led Semaglutide Prescribing Algorithm for Type 2 Diabetes and Chronic Kidney Disease: Research Letter Supplemental Material for Validation of a Pharmacist-Led Semaglutide Prescribing Algorithm for Type 2 Diabetes and Chronic Kidney Disease: Research Letter by Maneka Sheffield, Emma Phelan, Natalie Ratajczak, Marisa Battistella, Karthik Tennankore, Ayodele Odutayo, Steven Soroka, Penelope Poyah, Keigan More, Heather Naylor, Natalie Kennie-Kaulbach, Daniel Rainkie, Jo-Anne Wilson in Canadian Journal of Kidney Health and Disease.

Supplemental Material - Validation of a Pharmacist-Led Semaglutide Prescribing Algorithm for Type 2 Diabetes and Chronic Kidney Disease: Research Letter Supplemental Material for Validation of a Pharmacist-Led Semaglutide Prescribing Algorithm for Type 2 Diabetes and Chronic Kidney Disease: Research Letter by Maneka Sheffield, Emma Phelan, Natalie Ratajczak, Marisa Battistella, Karthik Tennankore, Ayodele Odutayo, Steven Soroka, Penelope Poyah, Keigan More, Heather Naylor, Natalie Kennie-Kaulbach, Daniel Rainkie, Jo-Anne WIlson in Canadian Journal of Kidney Health and Disease.

Supplemental Material - Validation of a Pharmacist-Led Semaglutide Prescribing Algorithm for Type 2 Diabetes and Chronic Kidney Disease: Research Letter Supplemental Material for Validation of a Pharmacist-Led Semaglutide Prescribing Algorithm for Type 2 Diabetes and Chronic Kidney Disease: Research Letter by Maneka Sheffield, Emma Phelan, Natalie Ratajczak, Marisa Battistella, Karthik Tennankore, Ayodele Odutayo, Steven Soroka, Penelope Poyah, Keigan More, Heather Naylor, Natalie Kennie-Kaulbach, Daniel Rainkie, Jo-Anne Wilson in Canadian Journal of Kidney Health and Disease.

Supplemental Material - Validation of a Pharmacist-Led Semaglutide Prescribing Algorithm for Type 2 Diabetes and Chronic Kidney Disease: Research Letter Supplemental Material for Validation of a Pharmacist-Led Semaglutide Prescribing Algorithm for Type 2 Diabetes and Chronic Kidney Disease: Research Letter byManeka Sheffield, Emma Phelan, Natalie Ratajczak, Marisa Battistella, Karthik Tennankore, Ayodele Odutayo, Steven Soroka, Penelope Poyah, Keigan More, Heather Naylor, Natalie Kennie-Kaulbach, Daniel Rainkie, Jo-Anne Wilson in Canadian Journal of Kidney Health and Disease.

## Data Availability

The authors confirm that the data supporting the findings of this study are available within the article.*

## ORCID iDs

Jo-Anne Wilson https://orcid.org/0000-0003-3423-5740

Maneka Sheffield https://orcid.org/0009-0007-8886-571X

Emma Phelan https://orcid.org/0009-0004-5285-753X

Marisa Battistella https://orcid.org/0000-0001-9456-4365

Karthik Tennankore https://orcid.org/0000-0002-7919-6709

Ayodele Odutayo https://orcid.org/0000-0001-8619-5010

Steven Soroka https://orcid.org/0000-0002-7278-1702

Penelope Poyah https://orcid.org/0000-0002-7575-2835

Keigan More https://orcid.org/0000-0003-4903-6680

Heather Naylor https://orcid.org/0009-0008-3801-8907

Natalie Kennie-Kaulbach https://orcid.org/0000-0002-4792-9365

Daniel Rainkie https://orcid.org/0000-0002-9744-1069
