# Supplementary material for: Validation of a Pharmacist-Led Semaglutide Prescribing Algorithm for Type 2 Diabetes and Chronic Kidney Disease: Research Letter
Source: Can J Kidney Health Dis. 2026 Aug 13;13:20543581261476643. doi: 10.1177/20543581261476643 (PMC13473795; doi:10.1177/20543581261476643)
Supplement: Supplementary file 1 [file sj-pdf-1-cjk-10.1177_20543581261476643.pdf]

Supplementary File 1: Evidence Tables for Semaglutide Algorithm Development

Table of Contents

GLUCAGON-LIKE PEPTIDE-RECEPTOR 1 AGONISTS: SEMAGLUTIDE 2

Evidence Table *2*

Dosing, Monitoring, Cost, & Coverage Table (GLP-1 RA) *4*

REFERENCES 4

Glucagon-Like Peptide-Receptor 1 Agonists: Semaglutide

Summary:
Patients with diabetes are at an increased risk of developing microvascular complications such as diabetic nephropathy, which is a leading cause of chronic kidney disease (CKD). Semaglutide is a glucagon-like peptide-1 receptor agonist (GLP-1RA) that offer significant benefits in the management of diabetic nephropathy that extend beyond blood sugar and weight control. Semaglutide (subcutaneous) reduces albuminuria, **slows progression of CKD**, thus **delaying end-stage kidney disease** and the need for renal replacement therapy (such as dialysis or kidney transplantation). In addition, semaglutide (subcutaneous) has demonstrated a **reduction in major adverse cardiovascular events (MACE)**, **risk of death from any cause** and a **reduced annual eGFR fall from baseline** compared to placebo. It is a pharmacotherapy pillar of care for people with CKD and type 2 diabetes.

Evidence Table

Trial	Study Design	Population	Intervention/ Comparator(s)	Outcomes	Results
**Flow** (The Effect of Semaglutide on Chronic Kidney Disease in Patients with T2D) (9)<br><br>2024	MC, Pb-C, DB, RCT	N = 3533<br>≥18year with T2DM (HbA1C≤10%) + max. tolerated ACEi/ARB x ≥4 weeks + CKD<br><br>CKD defined:<br>eGFR ≥50 & ≤75mL/min/1.73m^2 AND UACR >300 & < 5000 mg/g (34 & 565 mg/mmol)<br><br>OR<br>eGFR ≥25 & <50mL/min/1.73m^2 AND UACR >100 & <5000mg/g (11 & 565 mg/mmol)	Semaglutide (Ozempic®) 1 mg or Pb subcut. weekly<br><br>8-week escalation regimen: 0.25 mg x 4 weeks, then 0.5 mg x 4 weeks, then 1 mg per week	1° outcome= composite kidney failure (dialysis, transplantation, or eGFR < 15 ml/min/1.73m^2), ≥50% reduction from baseline in eGFR, death from kidney or CV causes<br><br>2° outcomes= MACE composite, all-cause mortality, death (non-CV, non-kidney), annual rate of eGFR change	**<u>1° outcome:</u>**<br>**Major kidney disease events:**<br>HR: = 0.76 (0.66-0.88), NNT = 20/3.4 years<br>**CV death**<br>HR = 0.71 (0.56 to 0.89), NNT = 34/3.4 years<br><br>**<u>2° outcomes:</u>**<br>**MACE Composite**<br>HR = 0.82 (0.68 to 0.98), NNT = 45/3.4 years<br>**All-cause mortality**<br>HR=0.8 (0.67 to 0.95), NNT = 39/3.4years<br>**Mean annual eGFR slope difference**<br>1.14 ml/min/1.73m2 p<0.001<br><br>**<u>Safety</u>**<br>**Serious Adverse Event**<br>Semaglutide 49.6% vs. Pb 53.8%<br>**Other:**<br>Eye Disorders: Semaglutide 53(3%) vs. PB 31 (1.7%)<br>Gastrointestinal disorders: Semaglutide 79 (4.5%) vs Pb

					20 (1.1%)<br>A1C Baseline to week 104: HR = - 0.81% (-0.9% - -0.72%),<br>Change in Weight from Baseline to week 104: HR= -4.1 kg (-4.56 - -3.65)
**Sustain-6** (Semaglutide and Cardiovascular Outcomes in Patients with T2D) (10)<br><br>2016	MC, Pb-C, DB, RCT	N=3297<br>>50 year with CVD, CHF (NYHA Class II or III), CKD stage 3 or higher.<br>OR<br>>60 y with at least 1 CV risk factor (e.g. persistent microalbuminuria (30-299 mg/g, 3.4-34mg/mmol) or proteinuria, hypertension and left ventricular hypertrophy, left ventricular systolic or diastolic dysfunction or ankle index less than 0.9)	Semaglutide (Ozempic) 0.5 mg or 1.0 mg Subcut. once weekly for Pb for 104 weeks	1° outcome = first occurrence of CV death, nonfatal MI, or nonfatal stroke.<br><br>2° outcome = expanded composite CV outcomes, retinopathy complications, and new or worsening nephropathy.	**<u>1° outcome:</u>**<br>**First occurrence of CV death, nonfatal MI, or nonfatal stroke overall:**<br>HR: = 0.74 (0.0.58-0.95), NNT= 44/2.1 years<br><br>**Nonfatal MI:**<br>HR=0.74 (0.51-1.08), NNT= 100/2.1 years<br><br>**Nonfatal Stroke:**<br>HR= 0.61 (0.34-0.99), NNT= 91/2.1 years<br><br>**<u>2° outcome:</u>**<br>New or worsening nephropathy:<br>Lower in Semaglutide HR = 0.64 (0.46-0.88), NNT = 44/2.1 years<br><br>**Retinopathy complications (Vitreous hemorrhage, blindness or conditions requiring treatment with an intravitreal agent or photocoagulation):**<br>HR=1.76 (1.11 - 2.78), NNH = 83/2.1 years<br><br><u>**Safety**</u><br>**Serious Adverse Event**<br>Semaglutide 33.6% vs. Pb 36.1%<br>**Other:**<br>Gastrointestinal disorders: Semaglutide 420 (52.1%) vs. Pb 290 (35.2%)

Abbreviations: 1° = primary; 2° = secondary; ACEi = angiotensin converting enzyme inhibitor; ARB = angiotensin II receptor blockers; CHF = chronic heart failure; CKD = chronic kidney disease; CV = cardiovascular; CVD = cardiovascular disease; DB= double-blind; eGFR = estimated glomerular filtration rate; HR = hazard ratio; MACE = major adverse cardiovascular events; MI = myocardial infarction; MC= multicenter; N =

number; NNT = number needed to treat; Pb = placebo; Pb-C = placebo-controlled; Subcut. = subcutaneous; RCT = randomized controlled trial; RRR = relative risk reduction; SC=subcutaneous; T2D = type 2 diabetes; UACR = urine albumin creatinine ratio; w. = weeks; y=years

Dosing, Monitoring, Cost, & Coverage Table (GLP-1 RA)

**Drug** (Brand)	**Dosing (Usual) (11-14)**	**Monitoring**	**Unit Price (15)**	**NS Formulary Coverage (15)**
**Semaglutide (Ozempic)**	**CPS, Canadian Product monograph & Lexicomp & Micromedex:**<br>Initial = 0.25 mg Subcut. weekly x 4 weeks, then 0.5 mg SC weekly x 4 weeks<br>Maintenance = 1 mg Subcut. weekly<br>Maximum = 2 mg SC weekly	A1C – Every 3 months then twice yearly once stable<br><br>Blood glucose – depending on goals of therapy<br><br>SCr – baseline and periodically	2 mg / 1.5 mL prefilled pen = 221.96<br>2 mg / 3 mL prefilled pen = 221.96<br>4 mg / 3 mL prefilled pen= 221.96	E = Exception<br><br>(For the treatment of type 2 diabetes in combination with metformin and a sulfonylurea, when diet and exercise plus dual therapy with metformin and a sulfonylurea do not achieve adequate glycemic control.)

Abbreviations: NS = Nova Scotia; SC = subcutaneous; SCr = serum creatinine

References

1. Tobe SW, Bajaj HS, Tangri N et al.. Chronic Kidney Disease in Diabetes: A Clinical Practice Guideline. *Can J Diabetes*. 2025 Mar;49(2):73-86.e14.

2. American Diabetes Association Professional Practice Committee. Diagnosis and Classification of Diabetes: Standards of Care in Diabetes-2024. *Diabetes Care*. 2024 Jan;47(Suppl 1):S20-S42.
3. American Diabetes Association Professional Practice Committee. Chronic Kidney Disease and Risk Management: Standards of Care in Diabetes-2024. *Diabetes Care*. 2024 Jan;47(Suppl 1):S219-S230.
4. Kidney Disease: Improving Global Outcomes (KDIGO) CKD Work Group. KDIGO 2024 Clinical Practice Guideline for the Evaluation and Management of Chronic Kidney Disease. *Kidney Int*. 2024;105(4S): S117–S314.
5. Kidney Disease: Improving Global Outcomes (KDIGO) Diabetes Work Group. KDIGO 2022 Clinical Practice Guideline for Diabetes Management in Chronic Kidney Disease. *Kidney Int.* 2022;102 (5S):S1–S127.
6. de Boer IH, Khunti K, Sadusky T et al. Diabetes Management in Chronic Kidney Disease: A Consensus Report by the American Diabetes Association (ADA) and Kidney Disease Improving Global Outcomes (KDIGO). *Diabetes Care*. 2022;45(12):3075-3090.
7. Chen JY, Hsu TW, Liu JH et al. Kidney and Cardiovascular Outcomes Among Patients With CKD Receiving GLP-1 Receptor Agonists: A Systematic Review and Meta-Analysis of Randomized Trials. *Am J Kidney Dis*. 2025 May;85(5):555-569.e1.
8. Natale P, Green SC, Tunnicliffe DJ et al. Glucagon- like peptide 1 (GLP- 1) receptor agonists for people with chronic kidney disease and diabetes. *Cochrane database Syst Rev.* 2025 Feb 18; 2(2):CD015849.
9. Perkovic V, Tuttle KR, Rossing P et al. Effects of Semaglutide on Chronic Kidney Disease in Patients with Type 2 Diabetes. *N Engl J Med*. 2024 Jul 11;391(2):109-121.
10. Marso SP, Bain SC, Consoli et al. Semaglutide and Cardiovascular Outcomes in Patients with Type 2 Diabetes. *N Engl J Med*. 2016 Nov 10;375(19):1834-1844.
11. Canadian Pharmacists Association. Semaglutide. In: *Compendium of Pharmaceuticals and Specialties* [Internet]. Ottawa (ON): Canadian Pharmacists Association; 2025 [cited 2026 Feb 17]. Available from: https://www.e-cps.ca
12. Lexicomp. Semaglutide. In: *Lexi-drugs online* [Internet]. Hudson (OH): Lexicomp, Inc.; c1978, 2024 [cited 2026 Feb 17]. https://online.lexi.com.
13. IBM Corporation. Semaglutide. In: Micromedex (In-Depth Answers) [Internet]. Greenwood Village (CO): IBM Corporation; 2026. [cited 2026 Feb 17]. Available from: https://www.micromedexsolutions.com.
14. Health Canada. Drug Product Database: Ozempic [Internet]. Ottawa (ON): Health Canada; 2024. [cited 2026 Feb 17]. Available from: https://www.canada.ca/en/health-canada/services/drugs-health-products/drug-products/drug-product-database.html
15. Nova Scotia Department of Health and Wellness. Nova Scotia Formulary [Internet]. Halifax (NS): Government of Nova Scotia; 2026 Available from: https://novascotia.ca/dhw/pharmacare/documents/formulary.pdf.
