# Supplementary material for: Validation of a Pharmacist-Led Semaglutide Prescribing Algorithm for Type 2 Diabetes and Chronic Kidney Disease: Research Letter
Source: Can J Kidney Health Dis. 2026 Aug 13;13:20543581261476643. doi: 10.1177/20543581261476643 (PMC13473795; doi:10.1177/20543581261476643)
Supplement: Supplementary file 2 [file sj-pdf-2-cjk-10.1177_20543581261476643.pdf]

Supplementary File 2. Two-Part Questionnaire for Algorithm Content and Face Validation

Round X: TWO-PART QUESTIONNAIRE - SEMAGLUTIDE ALGORITHM VALIDATION

Part of Previous Study: Optimizing Prescribing for Individuals with Diabetes and Chronic Kidney Disease through the Development and Validation of Algorithms for Community Pharmacists in Primary Care Clinics

The purpose of this questionnaire is to undergo algorithm content AND face validation for **Semaglutide algorithm only by community pharmacists**. The previously validated algorithms (e.g., ACEi/ARB, SGLT2i and Finerenone) are provided as a reference.
NOTE: The algorithms are designed to start first with ACEi/ARB to maximally tolerated dose, followed by SGLT2i with the 3rd or 4th agent as either finerenone or semaglutide based on patient factors and preferences.

Content validation refers to appropriateness, accuracy, completeness of the algorithm and **face validation** refers to clarity, comprehensibility, and appropriateness. Please follow the instructions provided and complete the questionnaire below based on your own expertise. Participation in this session is completely voluntary. If you have questions at any time, please feel free to contact the principal investigator, Dr. Jo-Anne Wilson.

Part ONE: Semaglutide Algorithm Content Validation
You have been provided with a copy of the semaglutide algorithm. The algorithm has been divided into individual items (A, B, C). Please rate each item on a scale of scale of 1 to 4 (1 – Unacceptable, remove; 2 – Unacceptable, major revision needed; 3 – Acceptable, with minor Revision; 4 – Acceptable, as is). For any item you do not find relevant, please provide a comment regarding why. A score of one or two indicates that the item should be removed or revised. Please circle (or hi-light) ratings. CHOOSE ONE ONLY

Item	Unacceptable, remove	Unacceptable, major revision needed	Acceptable, with minor revision	Acceptable, as is	Comments
**A**	1	2	3	4	
**B**	1	2	3	4	
**C**	1	2	3	4	

Additional Comments:

____

____

____

Part TWO: Semaglutide Algorithm Face Validation (Part A,B,C)

Algorithm to Assist Community Pharmacist Prescribing of **Semaglutide** in Community Pharmacy Primary Care Clinic or Primary Care Setting for Chronic Kidney Disease (eGFR 30 - 60 mL/min/1.73m²) in Type 2 Diabetes.

Please circle your level of agreement with each statement in the questionnaire. For any statement in which you do not rate "5" or "Strongly Agree", please provide a comment regarding why and/or how the algorithm may be improved in that aspect. A score of 1, 2, or 3 for any statement suggests that the aspect of the algorithm requires revision. CHOOSE ONE ONLY.

	Strongly Disagree	Disagree	Neutral	Agree	Strongly Agree	Comments
The algorithm is clear and understandable	1	2	3	4	5	
The algorithm uses appropriate language and wording	1	2	3	4	5	
The algorithm flows in a logical manner	1	2	3	4	5	
I would use this algorithm in my own practice	1	2	3	4	5	
I would be confident recommending the use of this algorithm	1	2	3	4	5	

Additional Comments:
