# Supplementary material for: Validation of a Pharmacist-Led Semaglutide Prescribing Algorithm for Type 2 Diabetes and Chronic Kidney Disease: Research Letter
Source: Can J Kidney Health Dis. 2026 Aug 13;13:20543581261476643. doi: 10.1177/20543581261476643 (PMC13473795; doi:10.1177/20543581261476643)
Supplement: Supplementary file 3 [file sj-pdf-3-cjk-10.1177_20543581261476643.pdf]

Supplementary File 3. Semaglutide Algorithm for Content and Face Validation

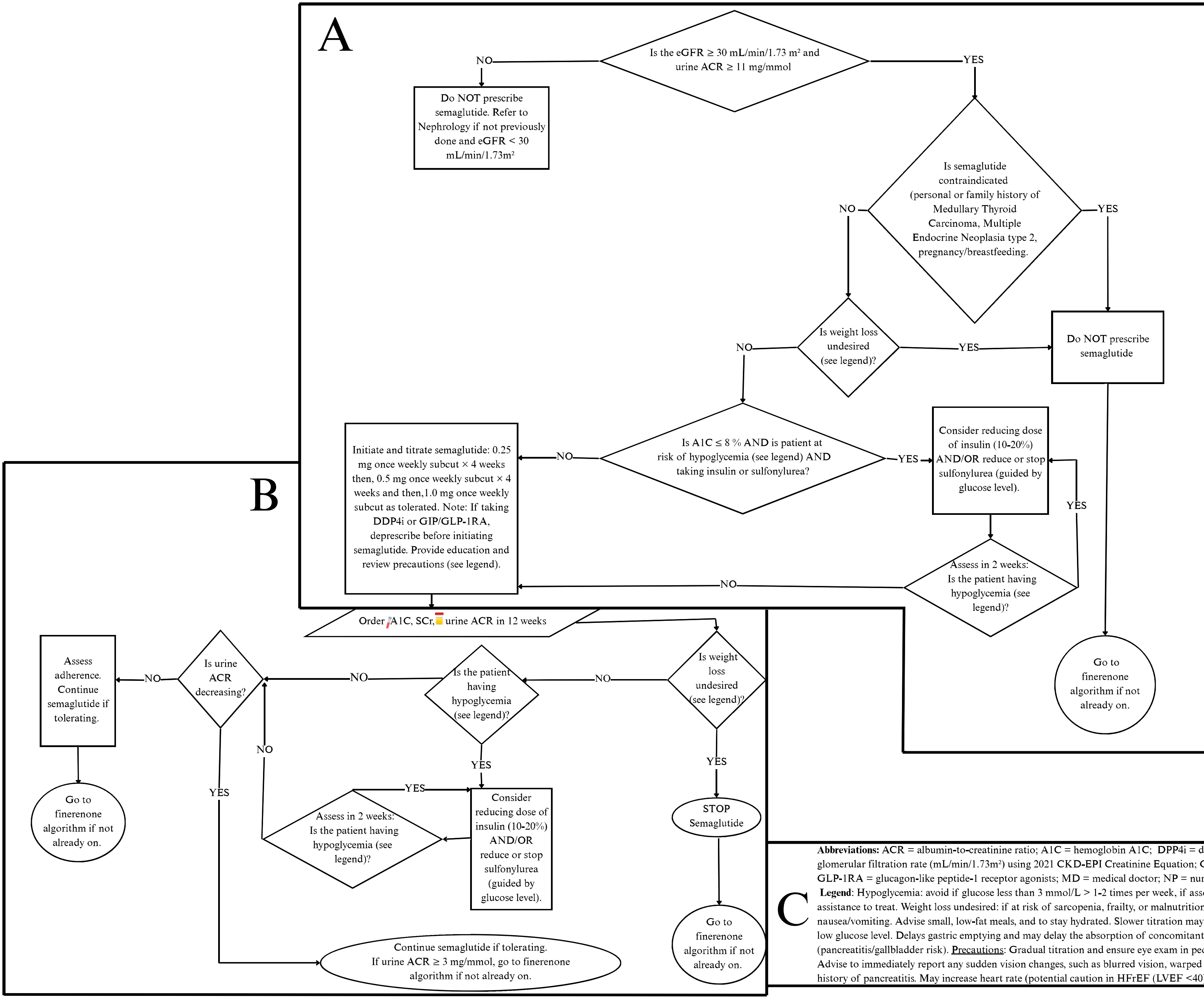

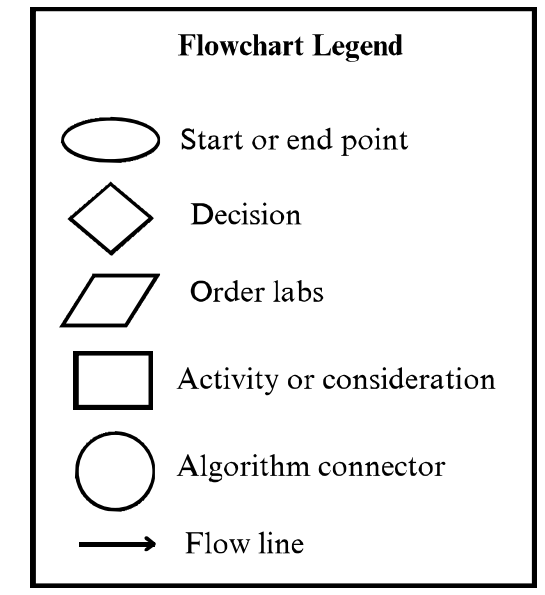

Disclaimer: Semaglutide pharmacist algorithm is for eGFR 30-60 mL/min/1.73m². However, semaglutide may be prescribed in individuals with an eGFR as low as 25 mL/min/1.73m². For eGFR < 30 mL/min/1.73m², consult NP/MD to refer to nephrology.

C

Abbreviations: ACR = albumin-to-creatinine ratio; A1C = hemoglobin A1C; DPP4i = dipeptidyl peptidase-4 inhibitors; eGFR = estimated glomerular filtration rate (mL/min/1.73m²) using 2021 CKD-EPI Creatinine Equation; GIP = glucose-dependent insulinotropic polypeptide; GLP-1RA = glucagon-like peptide-1 receptor agonists; MD = medical doctor; NP = nurse practitioner; SCr = serum creatinine,

Legend: Hypoglycemia: avoid if glucose less than 3 mmol/L > 1-2 times per week, if associated with neuroglycopenic symptoms, or if requires assistance to treat. Weight loss undesired: if at risk of sarcopenia, frailty, or malnutrition. Educate patient to start low, titrate slowly to limit nausea/vomiting. Advise small, low-fat meals, and to stay hydrated. Slower titration may be required to facilitate tolerability. Advise to report low glucose level. Delays gastric emptying and may delay the absorption of concomitant medications. Advise to report severe abdominal pain (pancreatitis/gallbladder risk). Precautions: Gradual titration and ensure eye exam in people with pre-existing retinopathy and high A1C. Advise to immediately report any sudden vision changes, such as blurred vision, warped vision, flashes, or floaters. Consider other therapies if history of pancreatitis. May increase heart rate (potential caution in HFrEF (LVEF <40)).
