# Supplementary material for: Validation of a Pharmacist-Led Semaglutide Prescribing Algorithm for Type 2 Diabetes and Chronic Kidney Disease: Research Letter
Source: Can J Kidney Health Dis. 2026 Aug 13;13:20543581261476643. doi: 10.1177/20543581261476643 (PMC13473795; doi:10.1177/20543581261476643)
Supplement: Supplementary file 4 [file sj-pdf-4-cjk-10.1177_20543581261476643.pdf]

Supplementary File 4: Nephrology Clinician and Community Pharmacist Content Validity Indices Scores

	EC 1	EC 2	EC 3	EC 4	EC 5	ECs in Agreement	I-CVI	S-CVI/Ave
**Round 1**								
A	1	1	0	1	1	5	0.8	0.93
B	1	1	1	1	1	5	1	
C	1	1	1	1	1	5	1	
**Round 2**								
A	0	1	0	1	1	3	0.6	0.73
B	0	1	0	1	1	3	0.6	
C	1	1	1	1	1	5	1	
**Round 3**								
A	1	1	1	1	1	5	1	1
B	1	1	1	1	1	5	1	
C	1	1	1	1	1	5	1	
						**Overall S-CVI Average (Rounds 1-3) =** 0.89		

	PhC 1	PhC 2	PhC 3	PhC 4	PhC 5	PhC 6	PhCs in Agreement	I-CVI	S-CVI/Ave
**Round 1**									
A	1	1	1	1	1	1	6	1	1
B	1	1	1	1	1	1	6	1	
C	1	1	1	1	1	1	6	1	
**Round 2**									
A	1	1	1	1	1	1	6	1	1
B	1	1	1	1	1	1	6	1	
C	1	1	1	1	1	1	6	1	
							**Overall S-CVI Average (Rounds 1-2) = 1**		

Key:

- Score of "1" = Item achieved a rating of 3 or 4
- Score of "0" = Item achieved a rating of 1 or 2
- I-CVI = # of pharmacists who gave a rating of 3 or 4 divided by the # of pharmacists
- S-CVI/Ave = The average of all I-CVI scores across all items per round

EC = Expert Clinician; I-CVI = item-level content validity index; S-CVI/Ave = scale-level content validity index based on the average method; PhC = Pharmacist

Supplementary File 4: Nephrology Clinician and Community Pharmacist Face Validity Indices Scores

**Expert Clinician**				
	**Round 1 (N = 5)**	**Round 2 (N = 5)**	**Round 3 (N = 5)**	**Overall**
**Q1**. The algorithm is clear and understandable	80%	80%	100%	87%
**Q2**. The algorithm uses appropriate language and wording	80%	80%	100%	87%
**Q3.** The algorithm flows in a logical manner	100%	80%	100%	93%
**Q4.** I would use this algorithm in my own practice	100%	60%	100%	87%
**Q5.** I would be confident recommending the use of this algorithm	100%	60%	100%	87%

**Community Pharmacist**			
	**Round 1 (N = 6)**	**Round 2 (N = 6)**	**Overall**
**Q1**. The algorithm is clear and understandable	100%	100%	100%
**Q2**. The algorithm uses appropriate language and wording	100%	100%	100%
**Q3.** The algorithm flows in a logical manner	100%	100%	100%
**Q4.** I would use this algorithm in my own practice	100%	100%	100%
**Q5.** I would be confident recommending the use of this algorithm	100%	100%	100%
