# Supplementary material for: Validation of a Pharmacist-Led Semaglutide Prescribing Algorithm for Type 2 Diabetes and Chronic Kidney Disease: Research Letter
Source: Can J Kidney Health Dis. 2026 Aug 13;13:20543581261476643. doi: 10.1177/20543581261476643 (PMC13473795; doi:10.1177/20543581261476643)
Supplement: Supplementary file 5 [file sj-pdf-5-cjk-10.1177_20543581261476643.pdf]

Supplementary File 5. Summary of Changes from Across Validation Rounds

Nephrology Clinician

Round 1 Changes Made

1. Under Part A: Changed initiation criteria from Urine ACR 3 mg/mmol to Urine ACR 11 mg/mmol- Based on the FLOW trial inclusion criteria.
2. Clarified wording “is urine ACR decreasing” from “is urine ACR improving” to align with interpretation of Urine ACR.
3. Clarified wording “is weight loss undesired” from “is there a concern with weight loss”. This was recommended as weight loss is a known positive effect from the drug, where the amount of weight loss that would be a concern varies based on patient and clinician. The wording was updated to support interpretation.
4. Updated arrows for better flow within the sequential algorithm.
5. Clarified wording “consider reducing dose of insulin (10-20%)” to “consider insulin dose reduction by 10-20%.”
6. Updated wording “assess in 2 weeks” decision point after consider insulin dose reduction to “is the patient having hypoglycemia” for clarity when assessing hypoglycemia.
7. Updated wording “is the patient at risk for hypoglycemia” to “is the patient having hypoglycemia” for clarity when assessing hypoglycemia.

Round 2 Changes Made

1. Added hypoglycemia guidance in Part B to assist the clinician when assessing hypoglycemia (“see legend” added to the “assess in 2 weeks” decision point)
2. Added to legend “levels of hypoglycemia: avoid if glucose less than 3 mmol/L > 1-2 times per week, if associated with neuroglycopenic symptoms or if requires assistance to treat” under hypoglycemia Part C
3. Added guidance to interpreting undesired weight loss in Part C (see legend” under weight loss decision point). Added to legend “weight loss undesired: at risk of sarcopenia, frailty, or malnutrition” in Part C.
4. Subheadings of “educate”, “precautions” and “monitoring” added for clarity to Part C.
5. HFrEF precaution added to Part C of legend “may increase heart rate (potential caution in HFrEF)”
6. Diabetic retinopathy precaution added to Part C of legend, “Gradual titration and ensure eye exam in people with pre-existing retinopathy and high A1C. Advise to immediately report any sudden vision changes, such as blurred vision, warped vision, flashes, or floaters.
7. GI precautions in Part C of legend updated to include “slower titration may be required to facilitate tolerability.”
8. Medication spacing clarified to “Space ≥ 1 hour before certain medications (ie. antibiotics, oral contraceptives, digoxin)” replacing “space ≥ 1 hour before antibiotics, oral contraceptives, digoxin.”
9. Changed Part B end point if Urine ACR is not decreasing, semaglutide continued if tolerated (previously “stop semaglutide if urine ACR not decreasing”). This was changed to support the use of semaglutide for other benefits, even if Urine ACR is not decreasing, as long as it is tolerated.
10. Changed hypoglycemia decision point in Part B to assess whether the patient is having hypoglycemia, from risk of hypoglycemia. This was updated as the patient will be on semaglutide at the time of assessment. The wording of glucose level was updated “guided by glucose level”, the words “plasma” and “blood” were removed from glucose level to indicate point of care glucose testing used.

Round 3 Changes Made

1. Minimal changes were made except under Part B where the layout was redesigned for better flow and clarity.
2. The second “is Urine ACR decreasing” decision point was removed and arrow was redirected into first “is Urine ACR decreasing” decision point.

Community Pharmacists

Round 1 Changes Made

1. Clarified medication spacing in Part C. Added “delays gastric emptying may reduce absorption of concomitant medications”. Removed reference to specific medications (oral contraceptives and digoxin).
2. Added guidance for patients with a history of pancreatitis in Part C “consider other therapies if history of pancreatitis”
3. Removed concurrent DDP4i and GIP/GLP-1RA from contraindications. Added direction to deprescribe DDP4i or GIP/GLP-1RA prior to semaglutide initiation under initiation and titration box (Part B).
4. Flow lines changed in Part B, under “is the patient having hypoglycemia”. If the patient is not having hypoglycemia in 2 weeks, the flow line connects to “is urine ACR decreasing” decision point. The decision point was moved to the left of the consideration to improve flow.
5. The second Urine ACR assessment decision point was removed in Part B. Semaglutide continued if tolerated, regardless of whether Urine ACR has decreased.

Round 2 Changes Made

1. Expanded education and changed formatting for improved clarity.
2. Moved abbreviations to the bottom of algorithm.
3. Added additional detail to hypoglycemia education: “Advise patient to report low glucose levels. Adjust antihyperglycemic agents as needed.”
4. Formatting and realignment of the algorithm for better visual representation.
