# Supplementary material for: Validation of a Pharmacist-Led Semaglutide Prescribing Algorithm for Type 2 Diabetes and Chronic Kidney Disease: Research Letter
Source: Can J Kidney Health Dis. 2026 Aug 13;13:20543581261476643. doi: 10.1177/20543581261476643 (PMC13473795; doi:10.1177/20543581261476643)
Supplement: Supplementary file 6 [file sj-pdf-6-cjk-10.1177_20543581261476643.pdf]

Supplementary File 6. Validated Semaglutide Algorithm within All Final Algorithms

Overview of the Management of Chronic Kidney Disease in Type 2 Diabetes

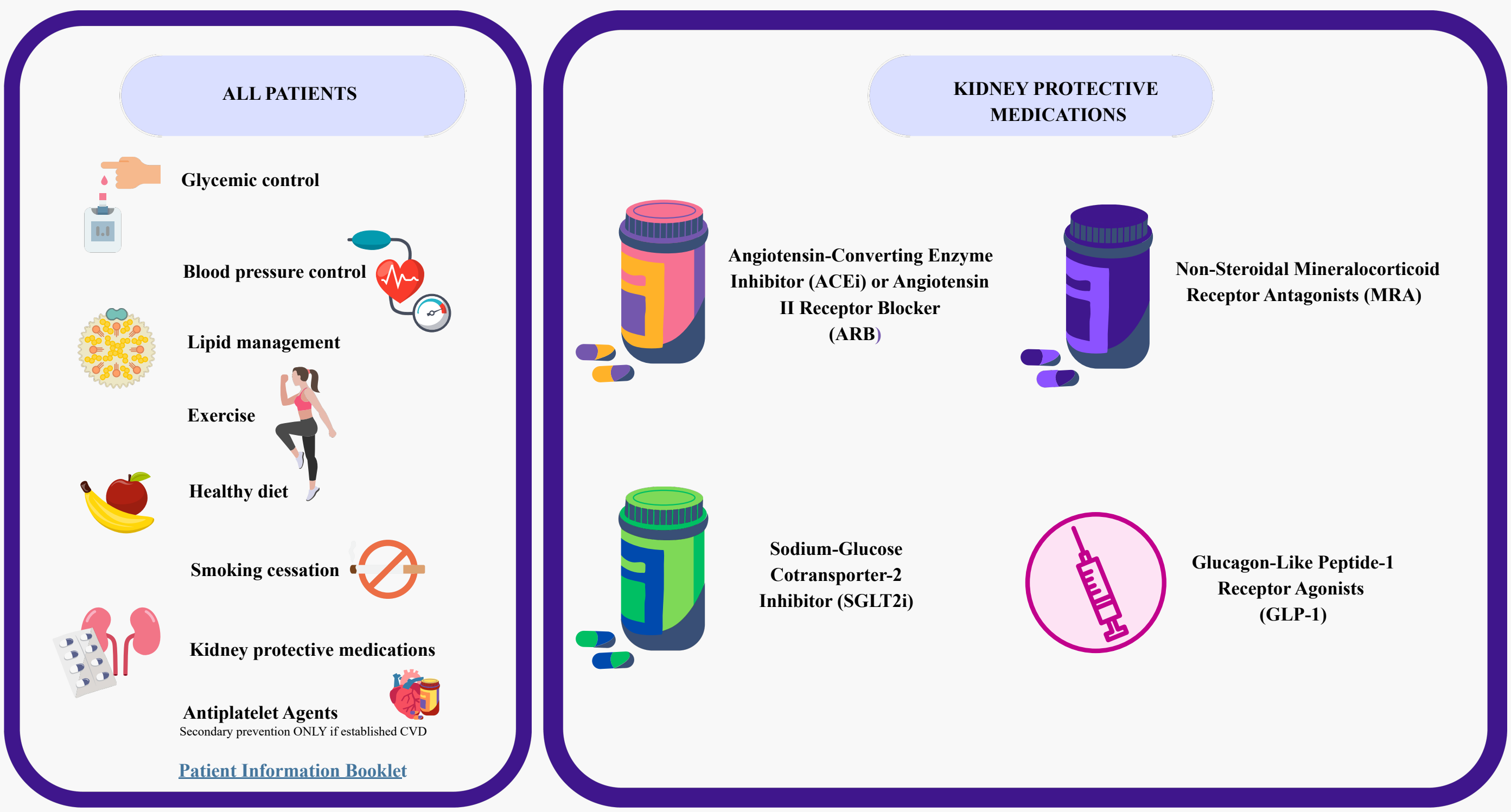

Abbreviations: ACEi = angiotensin-converting enzyme inhibitor; ARB = angiotensin II receptor blocker; CKD = chronic kidney disease; CVD = cardiovascular disease; RAS = renin-angiotensin system; SGLT2 inhibitor= sodium-glucose cotransporter-2 inhibitor; T2D = Type 2 diabetes;

Algorithm to Assist in Confirming Type 2 Diabetes (A) and Chronic Kidney Disease (B) Diagnosis in Community Pharmacy Primary Care Clinic or Primary Care Setting

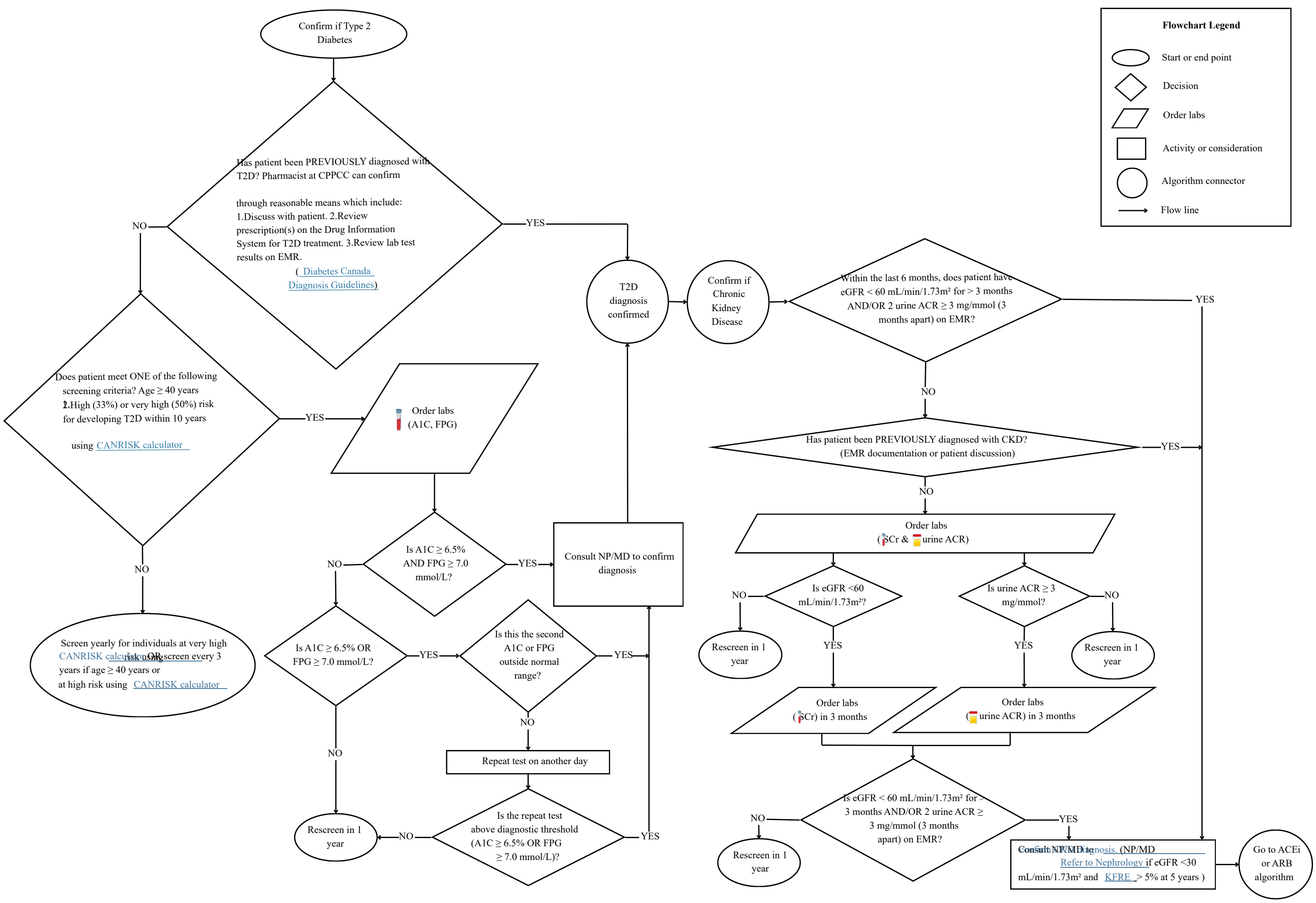

Abbreviations: A1C = glycated hemoglobin; ACEi = angiotensin-converting enzyme inhibitor; ACR = albumin-to-creatinine ratio; ARB = angiotensin II receptor blocker; CKD = chronic kidney disease; CPPCC = Community Pharmacy Primary Care Clinic; eGFR = estimated glomerular filtration rate (mL/min/1.73m²) using 2021 CKD-EPI Creatinine Equation; EMR=electronic medical record; FPG = fasting plasma glucose; KFRE = Kidney Failure Risk Equation; MD = medical doctor; NP = nurse practitioner; SCr = serum creatinine; T2D = type 2 diabetes

Algorithm to Assist Pharmacist Prescribing of Angiotensin-Converting Enzyme Inhibitors (ACEi) OR Angiotensin Receptor Blockers (ARB) in Community Pharmacy Primary Care Clinic or Primary Care Setting for Chronic Kidney Disease (eGFR 30-60 mL/min/1.73m²) in Type 2 Diabetes Part A: On ACEi or ARB with guidance for titration to maximally tolerated dose

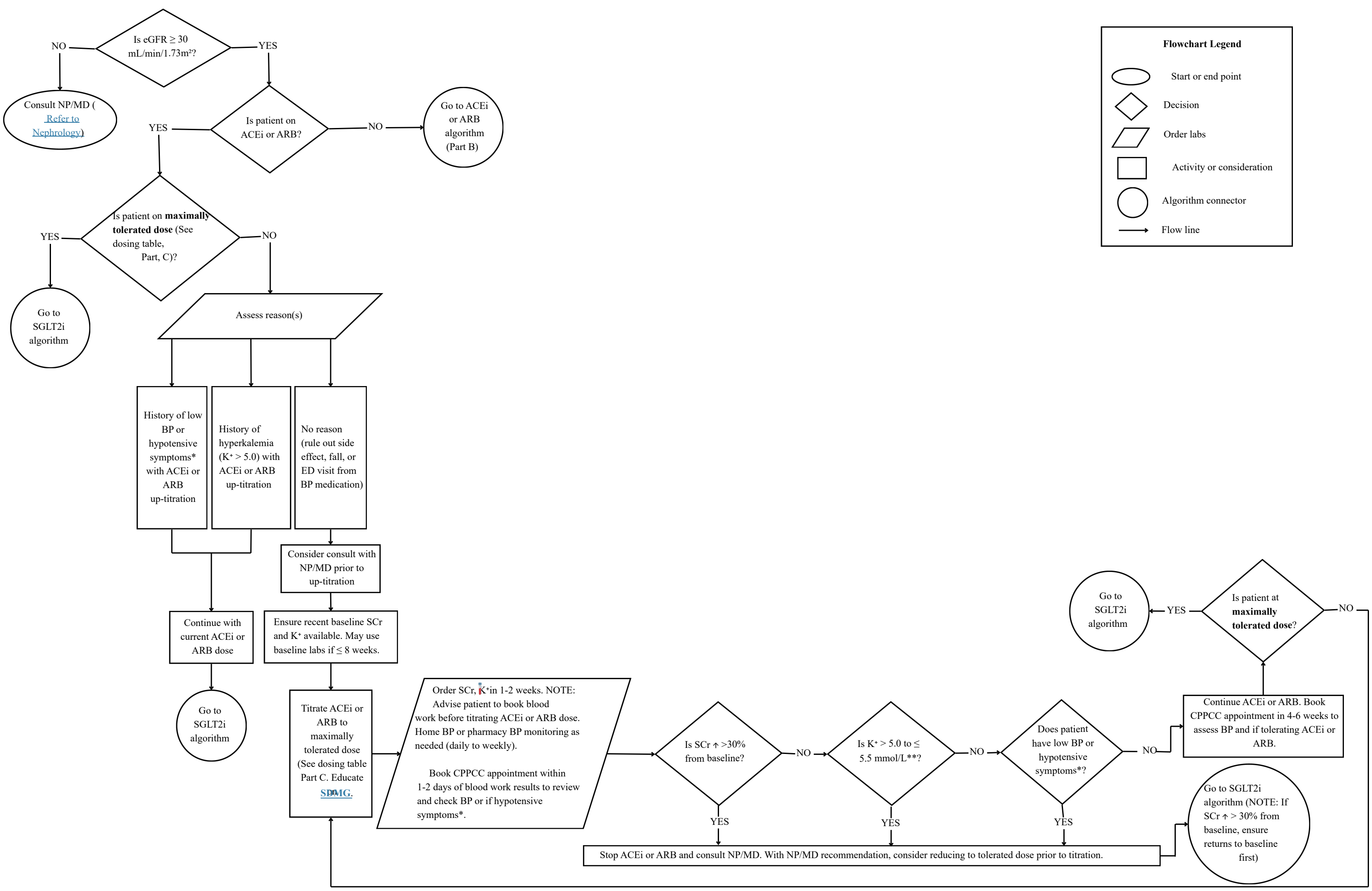

Abbreviations: ACEi = angiotensin-converting enzyme inhibitor; ARB = angiotensin II receptor blocker; BP = blood pressure [standardized BP]; ED = emergency department; eGFR = estimated glomerular filtration rate (mL/min/1.73m²) using 2021 CKD-EPI Creatinine Equation; K^+ = serum potassium; MD = medical doctor; NP = nurse practitioner; SCr = serum creatinine; **SDMG** = sick day medication guidance; SGLT2i = sodium-glucose cotransporter-2 inhibitor; T2D = Type 2 diabetes

Legend: Educate patient on triggers for SDMG and to temporarily hold ACEi or ARB if signs and symptoms of volume depletion such as diarrhea, vomiting, nausea, anorexia, new lightheadedness or weakness, lower weight or urine output, or increased thirst. Restart when back to normal eating and drinking and completely back to baseline; Advise regarding contraception in women. Consider restarting ACEi or ARB post-partum; *Hypotensive symptoms = light-headedness when standing from sitting or lying position, unsteadiness, dizziness, weakness, tired. **If K^+ > 5.5 mmol/L, stop ACEi or ARB. Consult NP/MD/Pharmacist Consult Service.

Algorithm to Assist Pharmacist Prescribing of Angiotensin-Converting Enzyme Inhibitors (ACEi) OR Angiotensin Receptor Blockers (ARB) in Community Pharmacy Primary Care Clinic or Primary Care Setting for Chronic Kidney Disease (eGFR 30-60 mL/min/1.73m²) in Type 2 Diabetes Part B: Not on ACEi or ARB with guidance for initiation and titration to maximally tolerated dose

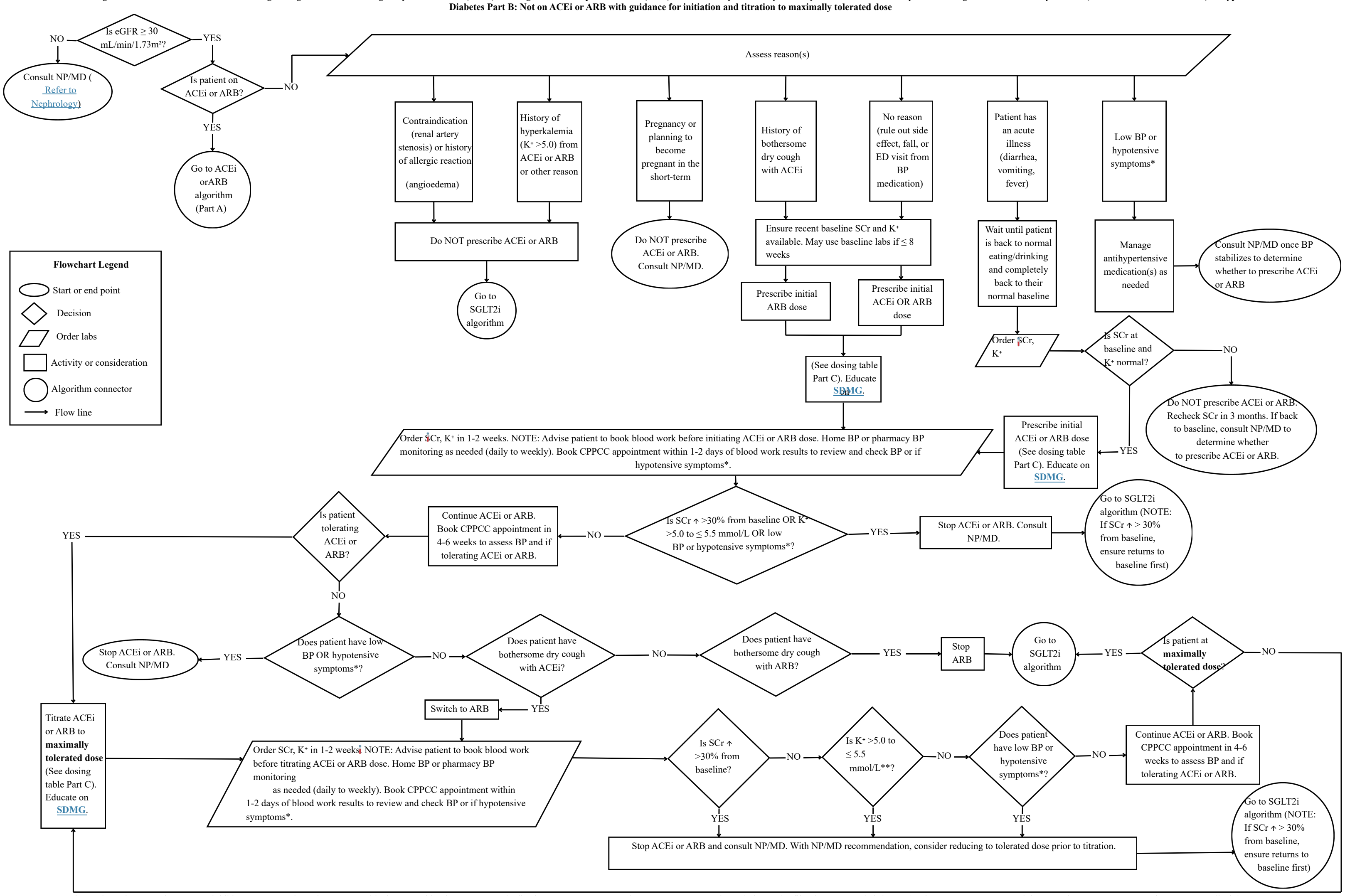

Abbreviations: ACEi = angiotensin-converting enzyme inhibitor; ARB = angiotensin II receptor blocker; BP = blood pressure [standardized BP]; ED = emergency department; eGFR = estimated glomerular filtration rate (mL/min/1.73m²) using 2021 CKD-EPI Creatinine Equation; K⁺ = serum potassium; MD = medical doctor; NP = nurse practitioner; SCr = serum creatinine; **SDMG** = sick day medication guidance; SGLT2i = sodium-glucose cotransporter-2 inhibitor; T2D = Type 2 diabetes

Legend: Educate patient on triggers for SDMG and to temporarily hold ACEi or ARB if signs and symptoms of volume depletion such as diarrhea, vomiting, nausea, anorexia, new lightheadedness or weakness, lower weight or urine output, or increased thirst. Restart when back to normal eating and drinking and completely back to baseline; Advise regarding contraception in women. Consider restarting ACEi or ARB post-partum; *Hypotensive symptoms = light-headedness when standing from sitting or lying position, unsteadiness, dizziness, weakness, tired. **If K⁺ > 5.5 mmol/L, stop ACEi or ARB. Consult NP/MD/Pharmacist Consult Service.

Part C: ACEi or ARB Dosing Table NOTE: Adults with T2D and CKD with either hypertension or albuminuria should receive an ACEi or ARB to delay progression of CKD

**ACEi**	**Initial Dose(s)***	**Maximum Dose**	**Strengths****
Lisinopril(Zestril)	5 -10 mg	40 mg	5 mg, 10 mg, 20 mg
Perindopril(Coversyl)	2-4 mg	16 mg	2 mg, 4 mg, 8 mg
Ramipril(Altace)	2.5-5 mg	10 mg	2.5 mg, 5 mg, 10 mg
Trandolapril(Mavik)	1 mg	4 mg	0.5 mg, 1mg, 2 mg, 4 mg
**ARB**	**Initial Dose(s)***	**Maximum Dose**	**Strengths****
Candesartan(Atacand)	8 mg	32 mg	4 mg, 8 mg,16 mg, 32 mg
Irbesartan(Avapro)	150 mg	300 mg	75 mg, 150 mg, 300 mg
Losartan(Cozaar)	25-50 mg	100 mg	25 mg, 50 mg, 100 mg
Olmesartan(Olmetec)	20 mg	40 mg	20 mg, 40 mg
Telmisartan(Micardis)	40 mg	80 mg	40 mg, 80 mg
Valsartan(Diovan)	80 -160 mg	320 mg	40 mg, 80 mg, 160 mg, 320 mg

*Lower starting doses may be necessary in older adults or if BP already at target.

**Drug Coverage (Seniors' Pharmacare, Community Services Pharmacare, Under 65-Long Term Care Pharmacare, & Family Pharmacare): All drugs/strengths listed in table EXCEPT candesartan 4 mg.

Educate patient on triggers for **sick day medication guidance (SDMG)** and to temporarily hold ACEi or ARB if signs and symptoms of volume depletion such as diarrhea, vomiting, nausea, anorexia, new lightheadedness or weakness, lower weight or urine output, or increased thirst. Restart when back to normal eating and drinking and completely back to baseline).

Maximally tolerated dose refers to the dose that is not associated with low BP or hypotensive symptoms OR an increase serum potassium > 5.0 OR acute kidney injury (serum creatinine rise > 30% from baseline). Increase dose to maximally tolerated dose at monthly intervals and/or based on individual patient parameters. Example 1: Individual on ramipril 5 mg daily and BP not at target --> is not on maximally tolerated dose. Example 2: Individual on ramipril 5 mg daily and BP below target -->is on maximally tolerated dose. Example 3: Individual on ramipril 5 mg daily and amlodipine 10 mg and BP at target ---> is not on maximally tolerated dose. Prioritize up-titration of ramipril to 10 mg daily and down-titration of amlodipine.

Doses obtained from CPS, Micromedex, Lexicomp, Canadian Drug Monographs.

Resources:

- Hypertension Canada
- Diabetes Canada
- KDIGO 2021 Blood Pressure and CKD
- KDIGO 2022 Diabetes and CKD
- KDIGO 2024 Clinical Practice Guidelines for Evaluation and Management of CKD

Algorithm to Assist Pharmacist Prescribing of Sodium-Glucose Cotransporter 2 Inhibitors (SGLT2i) in Community Pharmacy Primary Care Clinic or Primary Care Setting for Chronic Kidney Disease (eGFR 30-60 mL/min/1.73m²) in Type 2 Diabetes

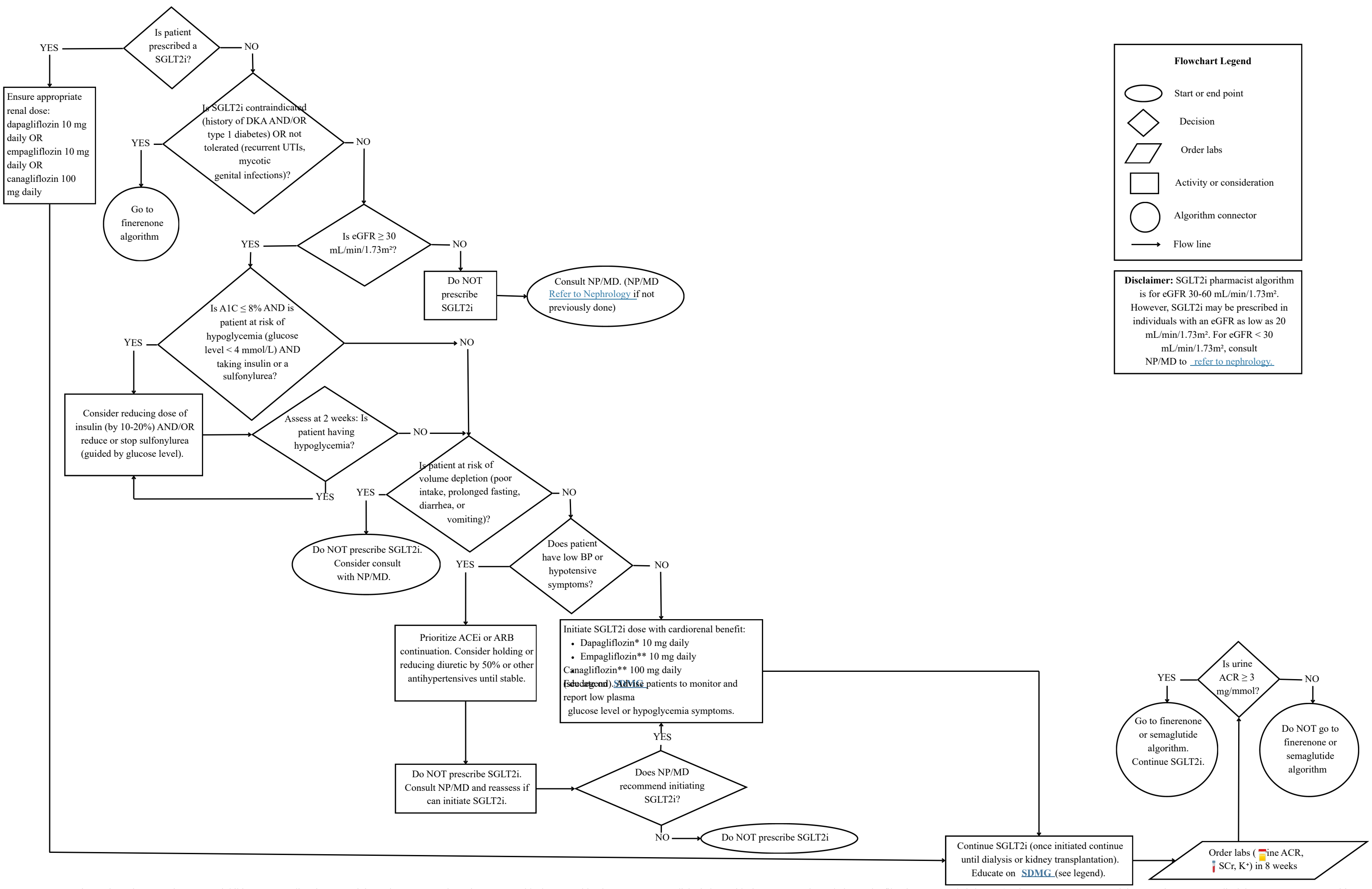

Abbreviations: ACEi = angiotensin-converting enzyme inhibitor; ACR = albumin-to-creatinine ratio; ARB = angiotensin II receptor blocker; BP = blood pressure; DKA = diabetic ketoacidosis; eGFR = estimated glomerular filtration rate (mL/min/1.73m²) using 2021 CKD-EPI Creatinine Equation; MD = medical doctor; NP = nurse practitioner; SCr = serum creatinine; **SDMG** = sick day medication guidance; SGLT2i = sodium-glucose cotransporter-2 inhibitor; UTI = urinary tract infection **Legend:** Volume depletion symptoms = dizziness when standing, muscle cramps, unable to urinate or reduced urination (not normal), tired or weak; hypotensive symptoms = light-headedness when standing from sitting or lying position, unsteadiness, dizziness, weakness, tired; Educate patient on triggers for **SDMG** and to temporarily hold SGLT2i if signs and symptoms of volume depletion such as diarrhea, vomiting, nausea, anorexia, new lightheadedness or weakness, lower weight or urine output, or increased thirst. Restart when back to normal eating and drinking and completely back to baseline; * = publicly funded; ** = publicly funded with criteria

Algorithm to Assist Pharmacist Prescribing of Finerenone in Community Pharmacy Primary Care Clinic or Primary Care Setting for Chronic Kidney Disease (eGFR 30-60 mL/min/1.73m²) in Type 2 Diabetes

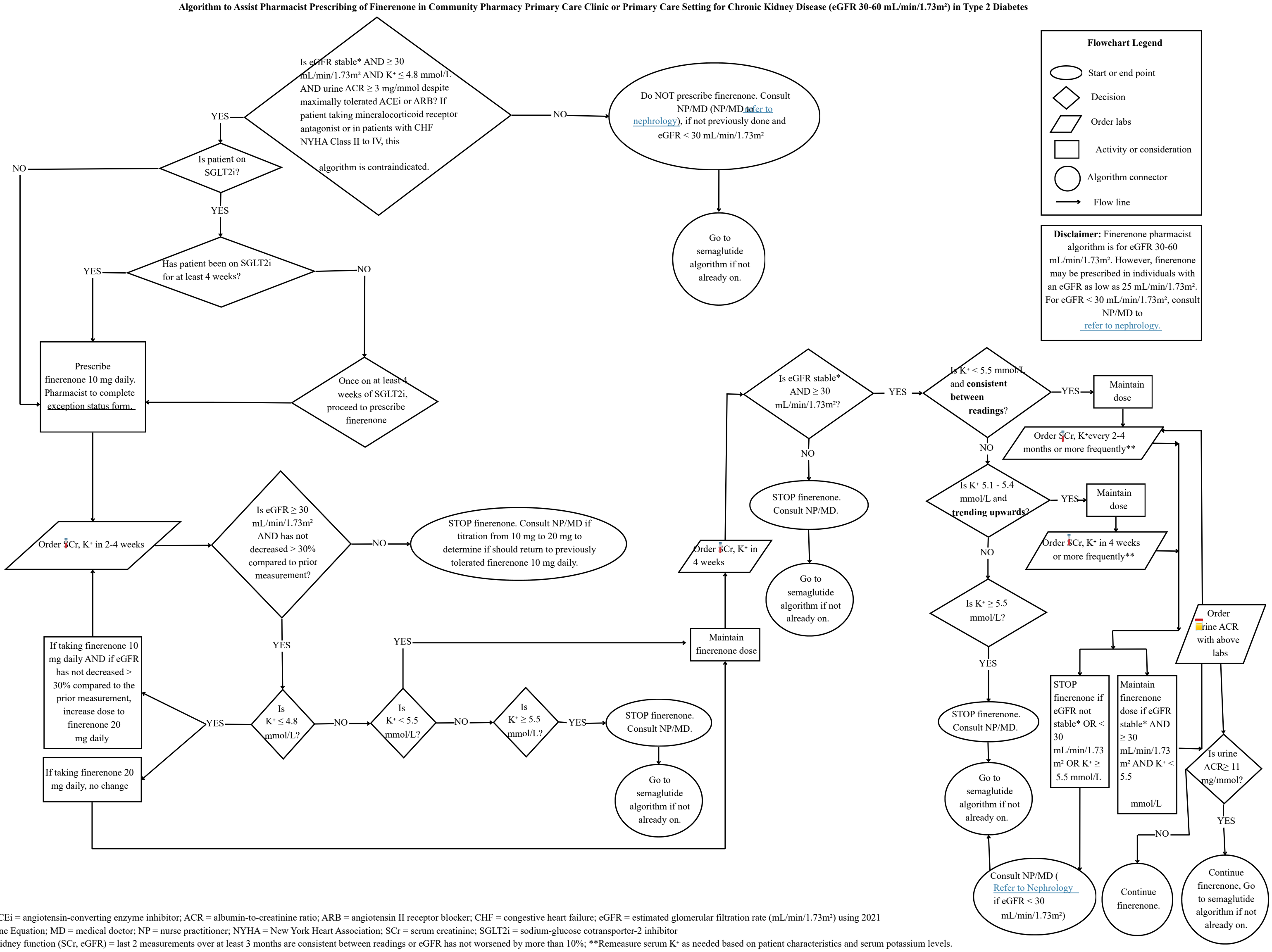

Abbreviations: ACEi = angiotensin-converting enzyme inhibitor; ACR = albumin-to-creatinine ratio; ARB = angiotensin II receptor blocker; CHF = congestive heart failure; eGFR = estimated glomerular filtration rate (mL/min/1.73m²) using 2021 CKD-EPI Creatinine Equation; MD = medical doctor; NP = nurse practitioner; NYHA = New York Heart Association; SCr = serum creatinine; SGLT2i = sodium-glucose cotransporter-2 inhibitor

Legend: *Stable kidney function (SCr, eGFR) = last 2 measurements over at least 3 months are consistent between readings or eGFR has not worsened by more than 10%; **Remeasure serum K⁺ as needed based on patient characteristics and serum potassium levels.

Algorithm to Assist Pharmacist Prescribing of Semaglutide in Community Pharmacy Primary Care Clinic or Primary Care Setting for Chronic Kidney Disease (eGFR 30-60 mL/min/1.73m²) in Type 2 Diabetes

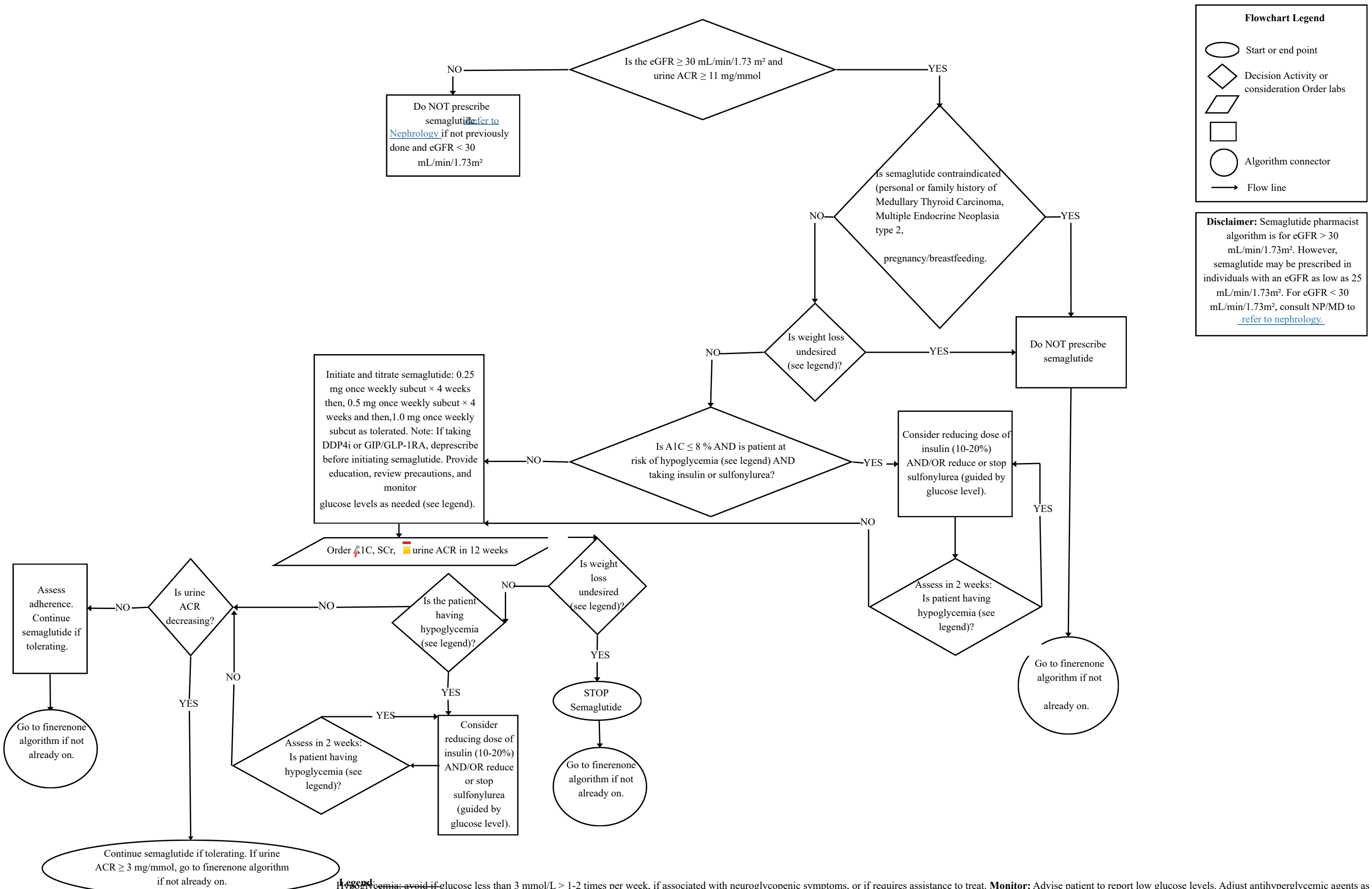

Legend: ~~Hypoglycemia: avoid if~~ glucose less than 3 mmol/L > 1-2 times per week, if associated with neuroglycopenic symptoms, or if requires assistance to treat. **Monitor:** Advise patient to report low glucose levels. Adjust antihyperglycemic agents as needed. Weight loss undes~~ired: if at risk of sarcope~~nia, frailty, or malnutrition. **Educate** patient to start low, titrate slowly to limit nausea/vomiting. Advise small, low-fat meals, and to stay hydrated. Slower titration may be required to facilitate tolerability. Delays gastric emptying and may delay the absorption of concomitant medications. Advise to report severe abdominal pain (pancreatitis/gallbladder risk). **Precautions**: Gradual titration and ensure eye exam in people with pre-existing retinopathy and high A1C. Advise to immediately report any sudden vision changes, such as blurred vision, warped vision, flashes, or floaters. Consider other therapies if history of pancreatitis. May increase heart rate (potential caution in HFrEF (LVEF <40)).

Abbreviations: ACR = albumin-to-creatinine ratio; A1C = hemoglobin A1C; DPP4i = dipeptidyl peptidase-4 inhibitors; eGFR = estimated glomerular filtration rate (mL/min/1.73m²) using 2021 CKD-EPI Creatinine Equation; GIP = glucose-dependent insulinotropic polypeptide; GLP-1RA = glucagon-like peptide-1 receptor agonists; MD = medical doctor; NP = nurse practitioner; SCr = serumcreatinine
